# Tuberculous arthritis of native joints – a systematic review and European Bone and Joint Infection Society workgroup report

**DOI:** 10.5194/jbji-8-189-2023

**Published:** 2023-08-28

**Authors:** Leonard C. Marais, Luan Nieuwoudt, Adisha Nansook, Aditya Menon, Natividad Benito

**Affiliations:** 1 Department of Orthopaedic Surgery, School of Clinical Medicine, University of KwaZulu-Natal, 719 Umbilo Road, Durban 4001, South Africa; 2 Department of Orthopaedic Surgery, School of Clinical Medicine, University of KwaZulu-Natal, Grey's Hospital, Townbush Road, Pietermaritzburg 3201, South Africa; 3 Division of Orthopaedic Surgery, Department of Surgical Sciences, Faculty of Medicine and Health Sciences, Stellenbosch University, Francie Van Zijl Avenue, Cape Town 7505, South Africa; 4 Department of Orthopaedics, P. D. Hinduja Hospital and Medical Research Centre, Mahim, Mumbai, India; 5 Infectious Diseases Unit, Hospital de la Santa Creu i Sant Pau, Institut d'Investigació Biomèdica Sant Pau (IIB), Universitat Autònoma de Barcelona, Barcelona, Spain; 6 Centro de Investigación Biomédica en Red de Enfermedades Infecciosas (CIBERINFEC), ISCIII, Madrid, Spain; 7 Centre for Clinical Research, The University of Queensland, Brisbane, Australia

## Abstract

**Introduction**: The aim of this systematic review was to assess the existing published data on the diagnosis and management of tuberculosis (TB) arthritis involving native joints in adults aged 18 years and older. **Methods**: This study was performed in accordance with the guidelines provided in the Preferred Reporting Items for Systematic reviews and Meta-Analysis extension for Scoping Reviews (PRISMA-ScR). **Results**: The systematic review of the literature yielded 20 data sources involving 573 patients from nine countries. There was considerable variation amongst the studies in terms of the approach to diagnosis and management. The diagnosis was mostly made by microbiological tissue culture. Medical management involved a median of 12 months of anti-tubercular treatment (interquartile range, IQR, of 8–16; range of 4–18 months). The duration of preoperative treatment ranged from 2 to 12 weeks. Surgery was performed on 87 % of patients and varied from arthroscopic debridement to complete synovectomies combined with total joint arthroplasty. The mean follow-up time of all studies was 26 months (range of 3–112 months). Recurrence rates were reported in most studies, with an overall average recurrence rate of approximately 7.4 % (35 of 475 cases). **Conclusions**: The current literature on TB arthritis highlights the need for the establishment of standardized guidelines for the confirmation of the diagnosis. Further research is needed to define the optimal approach to medical and surgical treatment. The role of early debridement in active TB arthritis needs to be explored further. Specifically, comparative studies are required to address questions around the use of medical treatment alone vs. in combination with surgical intervention.

## 1 Introduction

Primary tuberculosis (TB) infection is usually acquired via the inhalation of *Mycobacterium tuberculosis*. During primary infection, hematogenous dissemination may occur and lead to the seeding of bacilli at many sites, including bones and joints, although it is generally contained by cell-mediated immunity. Manifestations of bone and joint TB are more common during the reactivation of latent bacilli, often many years after the initial infection, but may also occur during primary infection (Hogan et al., 2017). The latter is more common in young patients in highly endemic areas, whereas the former occurs mainly in adults (Pigrau-Serrallach and Rodríguez-Pardo, 2013; Johansen et al., 2015). The most frequent form of skeletal TB is spondylitis (Pott's disease), followed by arthritis (Johansen et al., 2015; Mateo et al., 2007; Qian et al., 2018).

The most common presentation of tuberculous arthritis is that of chronic granulomatous monoarthritis (Berney et al., 1972). Two varieties of tuberculous arthritis have been described: the first originates in the synovium and joint capsule, whereas the second primarily affects bone. The latter can be further divided into pre-arthritic, arthritic and post-arthritic (inactive or quiescent) stages (Titov et al., 2004). In a series from Italy, the mean time from the onset of symptoms to the initiation of treatment was 216 d, which reflects the indolent nature of the disease (non-specific symptoms) and the difficulty in making the diagnosis (Mariconda et al., 2007). Tuberculous arthritis can mimic pyogenic, fungal, non-tuberculous mycobacterial or inflammatory arthritis (Huang et al., 2007). The most commonly affected sites are the weight-bearing joints, namely the hip, knee, foot and ankle regions, followed by the shoulder, elbow and wrist (Gardam and Lim, 2005).

A recent systematic review looked at the disability resulting from tuberculosis and found that musculoskeletal impairment was the third most common, after mental heath disorders and respiratory impairment (Alene et al., 2021). Early initiation of treatment is considered key to preventing permanent impairment. While cultures remain the standard confirmatory approach, the paucibacterial nature of the disease leads to low culture yields (Agashe et al., 2020). This has led to the use of more rapid and sensitive methods, including nucleic acid amplification tests (NAATs) such as polymerase chain reaction (PCR) (Mohanty et al., 2022). Despite the advances in our diagnostic abilities, a high index of suspicion is still required. In non-endemic areas, a TB diagnosis is often not considered, and this may result in unnecessary delays (Broderick et al., 2018). Many questions remain regarding the optimal management of TB arthritis. Most data relate to spinal TB, and there is little to guide treatment of extra-axial involvement, particularly in terms of the duration of therapy (Hogan et al., 2017).

In 2019, the European Bone and Joint Infection Society (EBJIS) initiated an interdisciplinary collaborative project in order to create a concise evidence-based clinical guideline for the management of septic arthritis of native joints (SANJO). The steering committee identified specific clinical dilemmas related to SANJO and created workgroups of international experts to address these. The guidelines emanating from this project have recently been published, and the publication summarizes the recommendations of the workgroups (Ravn et al., 2023). In the present report, the workgroup on TB arthritis of native joints presents the findings of a systematic review that aimed to assess the existing published data on the diagnosis and management of TB arthritis in adults.

## 2 Methods

This study was performed in accordance with the guidelines provided in the Preferred Reporting Items for Systematic reviews and Meta-Analysis extension for Scoping Reviews (PRISMA-ScR) (Tricco et al., 2018).

### 2.1 Research questions

Specifically, we focused on the following questions:

1. When should the diagnosis of arthritis caused by *Mycobacterium tuberculosis* be suspected?
2. What tests should be performed in patients with suspicion of native joint infection caused by *M. tuberculosis*?
3. What is the recommended antimicrobial therapy for arthritis due to *M. tuberculosis* and its optimal duration?
4. What is the recommended surgical management of native joint infection caused by *M. tuberculosis?*

### 2.2 Eligibility criteria

All original peer-reviewed studies at all levels of evidence were considered eligible for inclusion, including randomized and non-randomized control trials; prospective and retrospective cohort studies; case control studies; and analytical, cross-sectional and observational studies. Only studies published in English since 1970 were considered. Case series involving less than 10 patients, systematic reviews, narrative reviews, and non-clinical (basic science) or animal studies were excluded. We also excluded reports involving paediatric patients under the age of 18 years as well as all TB infections not involving a “peripheral native joint”, like TB of the spine, pubis, sacroiliac joint, temporomandibular joint and sternoclavicular joint as well as TB tenosynovitis or dactylitis. Periprosthetic joint infection secondary to TB and rheumatoid arthritis with secondary TB were also excluded.

### 2.3 Information sources

A systematic literature search of PubMed, Web of Science, Scopus and the Cochrane Library was undertaken on 2 March 2023. MeSH terms used, as per the 2020 MeSH descriptor data, were as follows: “Joint” AND “Tuberculosis” AND “Humans” (Mesh) AND “English” (lang).

### 2.4 Search strategy

**Figure 1:**
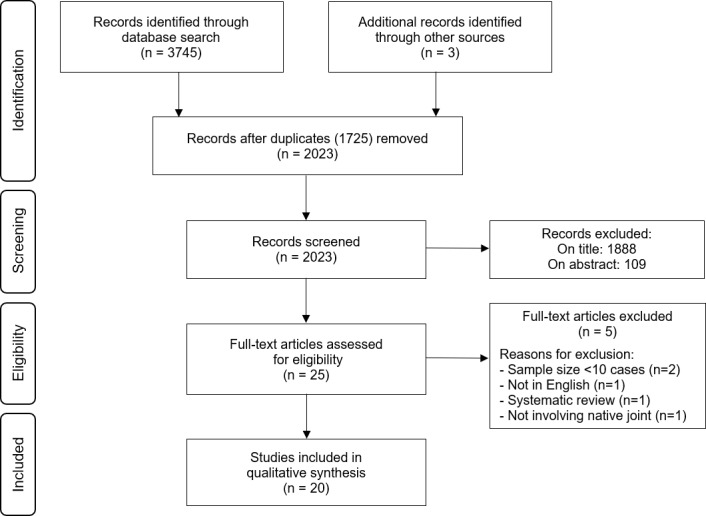
Flow diagram showing the selection of included studies.

The phenomena of interest was defined as intervention aimed at the diagnosis and/or management of tuberculosis of native joints in adult patients 18 years or older. After performing the literature search, articles were first excluded based on the title and abstract (Fig. 1). All remaining articles were assessed based on the full text whilst considering the inclusion/exclusion criteria. The selection was performed independently by two reviewers (Leonard C. Marais and Luan Nieuwoudt), and disagreements were resolved by consensus.

### 2.5 Data extraction

Data descriptors that were extracted included authors, country of origin, year of publication, study design, number of participants and outcomes of interest. In terms of the outcomes of interest, the following data were extracted from each study: anatomic location of infection, diagnostic methods, medical and surgical management, and the outcomes and follow-up period.

### 2.6 Synthesis of results

Results were reported according to PRISMA-ScR (Tricco et al., 2018). Due to the paucity of data and the lack of high-level evidence, a qualitative description of the data was performed in the form of a thematic analysis in accordance with our research questions. The level of evidence and strength of recommendations was assessed in accordance with the GRADE (Grading of Recommendations, Assessment, Development and Evaluations).

## 3 Results

The systematic review of the literature yielded 2023 potential sources, following deduplication, for screening. A description of the selection of the sources of evidence is depicted in Fig. 1. Following full-text review of 25 sources, 5 were excluded: 2 systematic reviews, 1 data source focused on total hip arthroplasty (not involving native joints), 1 publication in a language other than English and 2 series on extra-pulmonary TB that involved less than 10 cases of tuberculous arthritis. Thus, 20 data sources from nine countries were considered eligible for inclusion. The characteristics of the included studies are provided in Table 1.

**Table 1:** Characteristics of included sources of evidence and clinical features.

Author and	Country of	Sample	Patient	Previous or	Joint	Mono- vs.	Clinical
year ofpublication	origin	size ${}^{a}$	age ${}^{b}$	concomitant TB	involved	polyarticular	characteristics
Qian et al. (2018)	China	40	55.9 (25–83)	N/S	Multiple	No polyarticular cases	Knee ( n=11 ), hip ( n = 10), shoulder ( n = 7), elbow ( n = 6), ankle/foot ( n = 4) and wrist ( n = 2); average duration 6 months (range of 1–72)
Titov et al. (2004)	Russia	11	41.6 (17–72)	N/S	Multiple	No polyarticular cases	Active ( n = 8) and quiescent ( n = 3) knee TB, active ankle TB ( n = 1), and active elbow TB ( n = 1)
Huang et al. (2007)	Taiwan	51	58.6 (31–89)	10 % had previous pulmonary TB (PTB) (5 of 51)	Multiple	12 % (6 of 51) polyarticular cases	Knee ( n = 16), wrist ( n = 12), ankle ( n = 11), hip ( n = 10), shoulder ( n = 4), elbow ( n = 3) and foot ( n = 1); mean duration of symptoms 25 months (range of 1–180); 17 of 51 patients with active pulmonary TB
Sandher et al. (2007)	England	18	36.9 (12–93)	24 % had multiple sites of infection, including lungs, meninges, kidneys and gastrointestinal tract	Multiple	N/S	Hip ( n = 9), knee ( n = 5) and shoulder ( n = 4)
Lesić et al. (2010)	Serbia	25	50 % of patients 45–64 years of age	40 % had radiographic signs of either active or healed PTB	Multiple	N/S	Hip ( n = 18) and knee ( n = 7)
Al-Sayyad and Abumunaser (2011)	SaudiArabia	13	40 (17–70)	N/S	Multiple	No polyarticular cases	Hip ( n = 3), knee ( n = 3), elbow ( n = 3), ankle ( n = 2), wrist ( n = 1) and shoulder ( n = 1); mean duration of symptoms 24 months (range of 6–96)
Dhillon (2002)	India	74	23 (1–78)	12 % (9 of 74) had evidence of active or healed PTB or gastrointestinal tract (GIT) tuberculosis	Foot and ankle	No polyarticular cases	Foot and ankle TB (including non-articular TB); constitutional symptoms in 33 of 74 patients; duration of symptoms 2–22 months
Tang et al. (2007)	China	10	33 (28–56)	N/S	Foot and ankle	No polyarticular cases	“End-stage” TB ankle; duration of symptoms 8–23 months
Samuel et al. (2011)	India	11	38 (19–73)	Two patients (18 %) had concomitant miliary PTB and one had a psoas abscess	Ankle	No polyarticular cases	Active and quiescent TB involving the ankle ( n = 7 purely arthritic, but the remainder, n = 4, also had osseous involvement); duration of symptoms 1–36 months
Gursu et al. (2014)	Turkey	42	34.4 (7–85)	16 % had active PTB	Foot and ankle	No polyarticular cases	Arthritis ( n = 13), bone and joint ( n = 29), bone ( n = 29), and soft tissue ( n = 6); mean time from onset to diagnosis 26 months (range of 1 month–15 years)

**Table 1:** Continued.

Author and	Country of	Sample	Patient	Previous or	Joint	Mono- vs.	Clinical
year ofpublication	origin	size ${}^{a}$	age ${}^{b}$	concomitant TB	involved	polyarticular	characteristics
Duan and Yang (2019)	China	15	37.5 (8–70)	33 % had previous PTB (5 of 15)	Foot and ankle	No polyarticular cases	Active TB of the ankle; mean duration of symptoms 16 months (range of 3–36); all cases early arthritis without severe destruction
Kapukayaet al. (2006)	Turkey	11	28 (19–55)	No cases with active or previous PTB	Shoulder	No polyarticular cases	Duration of symptoms 3–24 months
Kim et al. (2001)	South Korea	56	38 (20–60)	N/S	Hip	No polyarticular cases	Inactive TB of the hip
Kumar (2015)	India	65	48 (29–65)	N/S	Hip	No polyarticular cases	Inactive TB of the hip joint; mean interval from completion of initial course of ATT to surgery 4 years (range of 2–6 years); excluded active TB
Zeng et al. (2015)	China	32	49.4 (24–79)	N/S	Hip	No polyarticular cases	Advanced TB arthritis of the hip; 11 patients presumed active with increased inflammatory marker
Liu et al. (2018)	China	40	46.8 (34–72)	N/S	Hip	No polyarticular cases	Average duration 4 years (range of 0.5–12)
Shen et al. (2010)	China	10	38.5 (14–56)	All patients' chest radiographs normal	Knee	No polyarticular cases	Active knee TB; median duration of symptoms 12 months (range of 4–36 months)
Habaxi et al. (2014)	China	10	40.6 (22–64)	N/S	Knee	No polyarticular cases	Active knee TB
Ozturkmenet al. (2014)	Turkey	12	56 ± 9.8	8 % had previous PTB (1 of 12)	Knee	No polyarticular cases	Advanced joint destruction and active TB; mean time from diagnosis to surgery 4 ± 1.5 months; patients with stage-1 and -2 tuberculosis as well as other sites of TB excluded
Tang et al. (2015)	China	26	43.7 (21–72)	N/S	Knee	No polyarticular cases	Active and quiescent TB of the knee; 15 patients with abscess around the knee and 7 with a sinus with mixed bacterial infections

${}^{a}$ Peripheral joints only, ${}^{b}$ Mean or median age in years (range), unless stated otherwise. The abbreviations used in the table are as follows: PTB – pulmonary tuberculosis; N/S – not stated; and GIT – gastrointestinal tract.

The included studies were highly heterogenous in terms of their inclusion criteria. In total, 573 patients with TB arthritis involving the extremities were included. In some studies, however, patients with a combination of bone and joint involvement were included, while other work did not clearly differentiate between those with pure joint or bone involvement. Most studies were observational and descriptive in nature, with one paper comparing the functional outcome following hip arthrodesis and arthroplasty (Liu et al., 2018).

### 3.1 Suspicion of TB arthritis

In the included studies, the clinical suspicion of TB arthritis was based on a combination of suggestive clinical and imaging findings. The preliminary clinical diagnosis was based on X-ray, computer tomography (CT) and/or magnetic resonance imaging (MRI) features; raised infection markers, such as C-reactive protein (CRP) and erythrocyte sedimentation rate (ESR); positive interferon-gamma release assays (IGRAs) or tuberculin skin tests; identification of acid-fast bacilli (AFB) on smear microscopy; and/or findings consistent with the diagnosis on histology (Table 2).

**Table 2:** Diagnostic findings in terms of imaging and laboratory investigations as well as the confirmatory test used in the included studies.

Author and year of publication	Imaging modalities and findings	Laboratory results	Confirmatory diagnostic tests
Qian et al. (2018)	Combination of X-ray, CT and MRI; no specific imaging findings stated	CRP, ESR, Hb, T-SPOT and sputum used; no specific results stated	Not stated; only two patients had biopsies
Titov et al. (2004)	X-rays used; specific X-ray findings not stated	Microscopy, cultures, PCR, anti-tuberculin, antibodies of blood and synovial fluid; no specific results stated	Tissue biopsy for culture and PCR (in some cases)
Huang et al. (2007)	Joint X-ray – bony destruction ( n = 9), bony fracture ( n = 7), osteoporotic change ( n = 6), osteolytic lesion ( n = 5), joint space narrowing ( n = 5), bony erosion ( n = 4), soft-tissue swelling ( n = 3), osteonecrosis (1) and cystic lesions ( n = 1); chest X-ray – evidence of active or old pulmonary TB in all patients, 26 of 51 (51.0 %); CT scan – revealed osteolytic lesions, destructive bones, sinus tract or soft-tissue masses in 9 of 10 cases; MRI – revealed arthritis with or without periarticular abscesses in three of four cases and a negative result in one case	48 of 51 had a synovial biopsy with 46 of 48 positive for mycobacterial infection (95.8 %)	At least one of the following confirmatory criteria is used: (1) *M. tuberculosis* isolated from synovial fluid or tissue biopsy specimen (positive in 84 % of cases), (2) histology of synovial tissue demonstrating caseating granuloma or granulomatous inflammation with positive AFB smear (positive in 96 % of cases), and (3) evidence of synovial granuloma with positive TB culture of the specimen other than synovial fluid or tissue (in 4 % of cases)
Sandher et al. (2007)	Joint X-rays – 43 of 79 (54 %) had evidence of bony destruction at infected site; further imaging was performed in the form of bone scans (4 of 79 cases) or CT/MRI scans (15 of 79 cases) when needed to evaluate extent of bone and soft-tissue involvement	27 of 79 (34 %) had positive bacteriology and histology, 16 of 79 (20 %) had positive bacteriology only, 15 of 79 (19 %) had positive histology only and 21 of 79 (27 %) had negative results treated on clinical suspicion	Positive bacteriology in 54 % of cases (test not specified); these included spinal cases, and TB arthritis was not reported separately
Lesić et al. (2010)	Joint X-ray – decalcification of the affected joint bones was the earliest X-ray sign of joint infection, and bone density later diminished with increased joint space; MRI – debris, thickening of synovial membranes and cartilage irregularities	31 % had positive culture from synovial fluid and 90 % had positive tissue culture (synovium); 45 % of positive bacteriology tuberculin skin tests (11 %) suggestive of TB	Diagnosis based on the Dolberg criteria; three diagnostics chosen from clinical features, radiological imaging, positive culture and response to anti-tuberculosis treatment; confirmatory tests – positive culture on synovial fluid (31 %) and/or positive culture on biopsy tissue (90 %)
Al-Sayyad and Abumunaser (2011)	Joint X-ray – soft-tissue swelling in seven patients (54 %), bone and joint destruction in four (31 %) and cysts and calcification in two (15 %), with computed tomography scan confirmation in involved cases; MRI – studies showed mainly joint effusion and synovitis	Bacteriology was positive in 9 patients (69 %); histopathology was positive in 12 patients (92 %)	Histology – 92 % positive; bacteriology (test not specified) – 69 % positive; remainder diagnosed on clinical grounds

**Table 2:** Continued.

Author andyear of publication	Imaging modalities and findings	Laboratory results	Confirmatory diagnostic tests
Dhillon (2002)	Cystic or osteoporotic changes were most seen; no other specific X-ray or CT findings mentioned	Anaemia – 19 of 74; ESR – raised in 74 of 74 at 25–100 mm h ${}^{-1}$ ; Mantoux test in 73 of 74	Synovial fluid and/or tissue biopsy for MCS and histology (note that a biopsy was only done if aspiration did not yield sufficient material)
Tang et al. (2007)	Joint X-ray and MRI – all had severe joint destruction and, eventually, sclerosis, fibrous ankylosis, central and peripheral erosions, various degrees of osteoporosis, obscure subcartilaginous bone plates or small amounts of articular hydrops, abscesses, bone chips, and articular cartilage damage	Positive PCR in 5 of 10 pre-op; positive PCR in 5 of 10 post-op	PCR and/or histology
Samuel et al. (2011)	Joint X-ray – all patients had periarticular osteopenia and articular cartilage destruction on the radiographs	Positive cultures in 8 of 16 (50 %); 8 of 16 diagnosed with histopathology only	MCS and histopathologic examination of tissue obtained by biopsy
Gursu et al. (2014)	Joint X-ray – focal erosion in 23 of 70 (32.8 %), infiltrative in 18 of 70 (25.7 %), spina ventosa in 6 of 70 (8.6 %) and cystic in 4 of 70 (5.7 %)	ESR mean of 41.3 mm h ${}^{-1}$ (range of 22–101); CRP mean of 31.7 mg L ${}^{-1}$ (range of 12–88); positive tuberculin test in 66 of 70 (95 %)	Culture of tissue obtained by biopsy
Duan andYang (2019)	Joint X-ray – not stated; MRI – suggested that all patients had preoperative effusion in the ankle articular cavity, accompanied by bone oedema of the distal radius and the talus	Histopathology and culture positive in 13 of 15	Culture and PCR of tissue obtained by biopsy; 87 % of cases confirmed by culture; PCR results not reported
Kapukaya et al. (2006)	Joint X-rays – Martin's classification used with one, two, four and four patients in stages 1–4, respectively	Mean ESR 22.1 ± 9.4 mm h ${}^{-1}$ (8 to 40 mm h ${}^{-1}$ ); Mantoux positive in 6 of 11; histopathology positive in 10 of 11; AFB positive in 1 of 11 on ZN staining	Culture was negative in all patients, but 10 of 11 patients had positive histology
Kim et al. (2001)	No specific X-ray, CT or MRI findings stated	Specific results not stated	PCR (since it has been available)
Kumar (2015)	All were diagnosed previously and not in this study	Histopathology and PCR positive in 0 of 65	Culture and PCR on biopsy tissue – all cultures negative and PCR not reported
Zeng et al. (2015)	Joint X-ray – clinical and radiologic features of these patients were consistent with stage-III and stage-IV osteoarticular tuberculosis of the hip	Histopathology positive in 32 of 32 (100 %)	Culture and histopathologic examination of tissue biopsy; culture was positive in 8 of 32 cases (25 %)

**Table 2:** Continued.

Author and year of publication	Imaging modalities and findings	Laboratory results	Confirmatory diagnostic tests
Liu et al. (2018)	MRI – swelling of surrounding tissues, narrowing of joint space, severe destruction of articular surface and adjacent bone, alteration of worm-eaten samples and formation of sequestrum	No specific results mentioned	Not specifically stated; diagnosis based on imaging and preoperative joint aspiration
Shen et al. (2010)	Joint X-ray – specific findings not stated	Histopathology and PCR positive in 10 of 10 (100 %); ESR – 10–37 mm h ${}^{-1}$ ; tuberculin test positive in 2 of 10 (20 %)	PCR (only two cases diagnosed preoperatively, remainder misdiagnosed preoperatively; diagnosis made postoperatively based on histological evaluation of tissue)
Habaxi et al. (2014)	Joint X-ray – narrowing of the knee joint; CT – joint destruction, purulent tissue formation around the joints, a low-density area and synovial hyperplasia.	ESR – 32–85 mm h ${}^{-1}$ ; histopathology positive in 10 of 10; AFB positive in 2 of 10 (20 %)	Histological assessment of tissue obtained at time of knee replacement
Ozturkmen et al. (2014)	Joint X-rays and MRI – no specific findings were specified for cases	Sputum – AFB and cultures negative in 12 of 12 (100 %); histopathology positive in 12 of 12	Culture (positive in five cases)
Tang et al. (2015)	Joint X-ray, CT and MRI – severe joint destruction, eventually leading to fibrous ankylosis, central and peripheral erosions, various degrees of osteoporosis, obscure subcartilaginous bone plates or small amounts of articular hydrops, abscesses, bone chips, and articular cartilage damage	Mean ESR – 46.2 ± 32.2 mm h ${}^{-1}$ ; mean CRP – 37.0 ± 35.9 mg L ${}^{-1}$ ; histopathology positive in 26 of 26 (100 %)	PCR or histological analysis of tissue specimen

The abbreviations used in the table are as follows: Hb – haemoglobin; ZN – Ziehl–Neelsen; AFB – acid-fast bacilli; WBC – white blood cell count; CRP – C-reactive protein; ESR – erythrocyte sedimentation rate; PCR – polymerase chain reaction; ELISA – enzyme-linked immunosorbent assay; MCS – microscopy, culture and sensitivity testing; and N/S – Not stated.

Suggestive clinical symptoms and signs of TB arthritis included monoarthritis with an indolent course (weeks to months or even years), joint pain, swelling, effusion, a draining sinus, restricted movement and/or deformity (Al-Sayyad and Abumunaser, 2011; Qian et al., 2018; Sandher et al., 2007; Huang et al., 2007; Duan and Yang, 2019; Kapukaya et al., 2006; Habaxi et al., 2014). Suggestive general clinical features included low-grade fever, night sweats, weight loss, coexisting pulmonary symptoms, previous TB, or history of living or having previously lived in an endemic area (Al-Sayyad and Abumunaser, 2011; Qian et al., 2018; Sandher et al., 2007; Duan and Yang, 2019; Kapukaya et al., 2006; Habaxi et al., 2014). Typical X-ray findings were periarticular osteopenia, subchondral erosions, periarticular cysts or fractures, narrowing of the joint space, and/or bony destruction or sclerosis. Other imaging investigations included CT (Al-Sayyad and Abumunaser, 2011; Habaxi et al., 2014; Tang et al., 2015) and MRI (Al-Sayyad and Abumunaser, 2011; Lesić et al., 2010; Tang et al., 2007; Habaxi et al., 2014; Ozturkmen et al., 2014).

Some studies included IGRAs. Qian et al. (2018) used the T-SPOT ELISPOT assay, which was positive in 42.6 % of patients. Dhillon and Nagi (2002) reported that ELISA (enzyme-linked immunosorbent assay) was used as a diagnostic adjunct and found it to be positive in 12 of 17 patients with foot or ankle TB (Dhillon and Nagi, 2002). The specific target analyte of their test was not stated. Tang et al. (2015) reported the use of blood antibody testing but not the results of the tests.

The results of AFB smears were reported in a small number of series. Huang et al. (2007) reported that 63 % of smears were performed on joint fluid aspirates or tissue samples. Conversely, Gursu et al. (2014) and Kapukaya et al. (2006) reported that only 0 % and 10 % of tests were positive, respectively. The results of the histopathological assessment of tissue samples were reported in nine studies and found to be positive in 87 %–100 % of cases (Huang et al., 2007; Al-Sayyad and Abumunaser, 2011; Tang et al., 2007; Gursu et al., 2014; Duan and Yang, 2019; Kapukaya et al., 2006; Zeng et al., 2015; Ozturkmen et al., 2014). However, in some of these series, histology served as a confirmatory and inclusion criterion.

### 3.2 Confirmation of diagnosis

The most common method used to confirm the diagnosis of TB is microbiological culture of tissue obtained by biopsy (Table 2) (Huang et al., 2007; Sandher et al., 2007; Al-Sayyad and Abumunaser, 2011; Dhillon and Nagi, 2002; Tang et al., 2007; Samuel et al., 2011; Gursu et al., 2014; Duan and Yang, 2019; Kapukaya et al., 2006; Kim et al., 2001; Kumar et al., 2015; Zeng et al., 2015; Shen et al., 2010; Habaxi et al., 2014; Ozturkmen et al., 2014; Tang et al., 2015; Liu et al., 2018). Overall, positive culture results ranged from 25 % to 100 % of cases. In some series, *M. tuberculosis* microbiological culture was also performed on synovial fluid aspirates (Huang et al., 2007; Dhillon and Nagi, 2002; Liu et al., 2018). Lešić et al. (2010) observed positive cultures in 31 % of joint fluid samples, whereas 90 % of tissue samples produced positive cultures. Huang et al. (2007) found positive histopathology in 96 % of synovial biopsies, while *M. tuberculosis* was cultured in 84 % of synovial tissue and/or fluid samples. AFB smear of synovial fluid or tissue was positive in only 63 % of cases. Al-Sayyad and Abumunaser (2011) reported positive histology in 92 % of cases and positive microbiology in 69 % of cases; however, neither the nature of the samples nor the method of analysis was noted. Zeng et al. (2015) reported positive histopathology in 100 % of their 32 patients, and positive *M. tuberculosis* culture on joint fluid and tissue was noted in 25 % of cases. In the studies that reported explicitly on the culture results (excluding studies including spine cases), the combined positive rate was 93 % (126 of 136 cases).

In some series, the diagnosis was confirmed through NAATs. PCR was used in several studies (Titov et al., 2004; Tang et al., 2007; Kim et al., 2001; Kumar et al., 2015; Duan and Yang, 2019). Kumar et al. (2015) reported that they performed PCR analysis on fluid and tissue obtained during surgery in all patients. The diagnostic accuracy of the test was not reported. The other studies did not report on the number of cases that were PCR-positive in comparison to culture.

In some series, the diagnostic criteria and methodology were not clearly defined and a combination of criteria were used, including clinical suspicion based on clinical history and physical examination, raised infection markers, Mantoux test results, radiological appearance (on X-rays, CT and/or MRI), response to anti-tuberculous therapy (ATT), microbiological analysis and/or histopathology (Qian et al., 2018; Titov et al., 2004; Sandher et al., 2007; Lesić et al., 2010). Lešić et al. (2010) established the diagnosis of TB via the application of the criteria put forth by Dolberg et al. (1991). According to this, the diagnosis of TB is made if any three of the following four criteria are met: typical clinical manifestation, radiological picture, positive *M. tuberculosis* culture or positive response to ATT.

### 3.3 Medical treatment

There was significant diversity in the approach to medical management demonstrated among the studies included in the data synthesis (Table 3). Most stated that they were following their respective national tuberculosis treatment guidelines. Overall, the medical management involved a median of 12 months of ATT (interquartile range, IQR, of 8–16; range of 4–18 months). In the studies that specified preoperative ATT, the median duration of preoperative ATT was 3 weeks (IQR of 2–4; range of 1–4 weeks) and the postoperative median duration was 12 months (IQR of 6–16; range of 2–18 months). There was considerable variation in the ATT agents used amongst the included studies. In collating these results, five groups emerged according to the anatomical site of infection, consisting of hip, knee, foot and ankle, shoulder, and mixed cohorts.

**Table 3:** Management strategies employed and outcomes reported in the included sources of evidence.

Author and year of publication	Involved joints	Medical management	Surgical management	Outcome	Follow-up
Qian et al. (2018)	Mixed cohort	Median duration of ATT 8–9 months, agents not specified; resistance patterns – H = 28 %, R = 17 %, E = 12 %, S = 18 % and MDR = 2 %	Overall, 57 % received surgery (including spine cases); joint arthrolysis for limited range of motion; arthroplasty used for joint destruction	82 % cure rate at 1 year; mortality 4 % at 1 year (includes spinal cases)	Mean 14 months (range of 12–24)
Titov et al. (2004)	Mixed cohort	Highly varied; most cases had 1 month of treatment pre-op and 2 months of treatment post-op	All patient had arthroscopic debridement (two converted to open debridement)	Significant functional improvement; no cases of reactivation or recurrence	15–42 months
Huang et al. (2007)	Mixed cohort	Not clearly specified; mean duration 15 months; resistance patterns – Z = 2 %, E = 2 %, S = 2 % and MDR = 4 %	92 % had surgical synovectomy	Relapse (8 of 36; 22 %)	Mean 32 months (range of 3–110); 30 % lost to follow-up
Sandher et al. (2007)	Mixed cohort	HRE ± Z (2 months) followed by HR (4 months); two cases with TB meningitis and 12 months of ATT; 9 % drug intolerance, regime modified; 7 % isoniazid resistance	Not specifically stated for non-spinal cases	No recurrences; no patients that underwent an open or arthroscopic washout required further surgical intervention	N/S
Lesić et al. (2010)	Mixed cohort	HREZ (2 months) followed by HR (4–12 months)	25 % surgical intervention; not stated separately for joint involvement	95 % favourable outcome; not stated separately for joint involvement	N/S
Al-Sayyad and Abumunaser (2011)	Mixed cohort	HREZ (18 months)	Synovectomy ( n = 12); arthrodesis ( n = 1)	No recurrence	1–10 years
Dhillon and Nagi (2002)	Foot and ankle	HREZ ± S (2–4 months) followed by two drugs (12–14 months)	Triple arthrodesis ( n = 3); talonavicular fusion ( n = 1); debridement ( n = 4)	Painful rocker bottom foot ( n = 2); painful flat foot ( n = 1)	N/S
Tang et al. (2007)	Ankle	ATT for ≥ 3 weeks pre-op until ESR normal or stable; post-op HRE (12–18 months) plus Z (for 6 months) plus S (1–3 months)	All had arthroscopically assisted ankle fusion	All fusions united and no recurrences	Mean 23 months (range of 6–46)
Samuel et al. (2011)	Foot and ankle	HREZ (2 months) followed by two drugs (4–16 months)	Synovectomy ( n = 2); ankle arthrodesis ( n = 4); tibiocalcaneal fusion ( n = 1)	Ankle arthrodesis healed (3 of 3); synovectomy with healing (1 of 1); tibiocalcaneal fusion resulted in forefoot equines; two patients lost to follow-up	Median 10 months (range of 9–39)

**Table 3:** Continued.

Author andyear of publication	Involved joints	Medical management	Surgical management	Outcome	Follow-up
Gursu et al. (2014)	Foot and ankle	Three- or four-drug ATT for a mean of 8 months (range of 3–17)	Soft-tissue debridement (40 %), bone debridement (34 %), fistulectomy (13 %), arthrodesis (7 %), synovectomy (6 %) and sequestrectomy (9 %)	Overall, 26 % recurrence (including osseous TB)	Mean 22 months (range of 8–30)
Duan and Yang (2019)	Ankle	Preoperative – HREZ ( ≥ 2–3 weeks until ESR decreased or became normal); postoperative – HREZ (3 months) followed by HRZ (3 months) and then HR (12 months); total treatment 18 months	Arthroscopic biopsy, synovectomy and debridement	No recurrence; no severe complications observed	≥24 months
Kapukaya et al. (2006)	Shoulder	HREZ (2 months) followed by HR (10 months)	Debridement ( n = 2); arthrodesis ( n = 1)	Five cases had a painless and mobile shoulder, three had mildly restricted shoulder motion without pain, and three had limited motion; no recurrence	Mean 29 months (range of 22–52)
Kim et al. (2001)	Hip	HRE (6–9 months)	Total hip replacement – < 1992 cemented ( n = 41) and ≥1992 uncemented ( n = 15)	Recurrence rate 9 %; one PJI	11–28 years
Kumar (2015)	Hip	HREZ (started 1 week prior to surgery and continued for 2 months); HR for 4 months thereafter	All patients underwent cementless total hip replacement	Recurrence in two cases; no other complications; no evidence of implant loosening	Mean 8 years (range of 6–11)
Zeng et al. (2015)	Hip	Preoperative – HREZ started 2 weeks prior to surgery (in 11 cases with raised inflammatory markers); postoperative – HREZ (12 months)	All patients underwent debridement and cementless total hip replacement	No reactivation; no implant loosening; no PJI or dislocations	Mean 4 years (range of 2–7)
Liu et al. (2018)	Hip	Preoperative – HRES (4 weeks and until ESR < 60 mm h ${}^{-1}$ and CRP < 35 mg L ${}^{-1}$ ); postoperative – HRES (12 months)	Debridement and total hip arthrodesis ( n = 18); debridement and total hip arthroplasty ( n = 22)	No reactivation; no complications in arthroplasty group; one non-union and one delayed union in arthrodesis group; postoperative pain and function superior in arthroplasty group	6–18 months
Shen et al. (2010)	Knee	Postoperative – HRE (8 months); in addition, intra-articular injection of 100 mg isoniazid every third day for 1 month after surgery	All had arthroscopic debridement and synovectomy	Significant functional improvement following surgery; no relapses	6–36 months

**Table 3:** Continued.

Author and year of publication	Involved joints	Medical management	Surgical management	Outcome	Follow-up
Habaxi et al. (2014)	Knee	“Quadruple” ATT started 2–4 weeks preoperatively with aim to achieve ESR < 40 mm h ${}^{-1}$ ; ATT agents used not stated; authors recommend 12–18 months of anti-tuberculous treatment	All patients had debridement and total knee arthroplasty (following 1 month of TB treatment and ESR < 40 mm h ${}^{-1}$ )	One recurrence (out of eight cases that were followed up)	Mean 14 months (range of 6–28)
Ozturkmen et al. (2014)	Knee	Postoperative – HREZ for 12 months; in three patients, CRP and ESR remained elevated at 6 months, and they received 18 months of ATT	All patients had debridement and two-stage total knee arthroplasty	No reactivation; no evidence of implant loosening; two cases showed radiolucent side around tibial component	Mean 6.1 ± 1.8 years
Tang et al. (2015)	Knee	Preoperative – HRE ( > 3 weeks until ESR became stable or decreased); postoperative – HRE (12 months or until inflammatory markers normal on at least three occasions); streptomycin added for first months	Open debridement and knee arthrodesis (single stage with transarticular screws and external fixator); 14 patients had prior arthroscopic ( n = 11) or open ( n = 3) debridement of the knee at the time of diagnosis	All cases fused at 14 months; mean time to radiologic union 5.6 months; mean leg length discrepancy 2.8 cm; one recurrence	Mean 5.5 years

The abbreviations used in the table are as follows: H – isoniazid; R – rifampin; E – ethambutol; Z – pyrazinamide; S – streptomycin; MDR – multidrug resistant; CRP – C-reactive protein; ESR – erythrocyte sedimentation rate; PJI – periprosthetic joint infection; and N/S – not stated.

The four studies on TB of the hip reported preoperative medical treatment consisting of a four-drug combination containing isoniazid (H), rifampicin (R), ethambutol (E), and pyrazinamide (Z) or streptomycin (S) for 1–4 weeks. Postoperatively, this regime was continued for between 6 and 12 months, most favouring not to de-escalate to two-drug regimens (three out of four series). Kim et al. (2011) reported on 56 hips with an 11- to 28-year follow-up period (Kim et al., 2001). Their typical preoperative treatment period was 3 months, and the postoperative treatment comprised 6 months with a three-drug (HRE) combination. Kumar et al. (2015) had a 1-week preoperative and a 2-month postoperative HREZ regimen, de-escalating to HR for a further 4 months. Zeng et al. (2015) and Liu et al. (2018) followed a similar 2 or 4 week preoperative regimen of HREZ or HRES and both continued for 12 months postoperatively using the same drugs.

Four studies focused on TB of the knee joint (Shen et al., 2010; Ozturkmen et al., 2014; Tang et al., 2015; Habaxi et al., 2014). Medical treatment followed a similar trend with preoperative three-drug (HRZ) or four-drug (HREZ) regimes for a period of 2–4 weeks, followed by the same drugs postoperatively for 8–18 months. Shen et al. (2010) reported 10 cases that all underwent arthroscopic debridement and an 8-month postoperative HRE drug administration. Ozturkmen et al. (2104) employed only postoperative ATT involving a 12-month HREZ protocol, while Tang et al. (2015) used HRE ± S for 3 weeks pre-op and 12 months post-op. Habaxi et al. (2014) was unclear on the exact anti-tubercular agents that were used, but they recommended 12–18 months of treatment in total. In their series, ATT was given for 2–4 weeks prior to arthroplasty, aiming for an ESR < 40 mm h ${}^{-1}$ .

In studies involving TB of the foot and/or ankle, a lower proportion of patients received surgery (Dhillon and Nagi, 2002; Tang et al., 2007; Samuel et al., 2011; Gursu et al., 2014). Preoperative treatment periods were between 8 and 12 weeks, consisting of four-drug combinations, in four out of five studies. This was ensued by two- to four-drug combinations for between 4 and 18 months post-op (median 11 months). In Gursu et al. (2014), the mean treatment duration was 8 months (range of 3–17 months) utilizing a three- or four-drug regimen.

One study (Kupukaya et al., 2006) investigating TB of the shoulder also used a four-drug (HREZ) regime for 8 weeks before surgery, switching to a two-drug (HR) 10-month strategy after debridement or fusion surgery.

Medical treatment extrapolation from the mixed-cohort studies was more difficult, with most studies not clearly specifying their exact protocols (Qian et al., 2018; Titov et al., 2004; Huang et al., 2007; Al-Sayyad and Abumunaser, 2011). The studies that did state their chemotherapy regimens favoured 2 months of three or four drugs (HRE or HREZ, respectively) pre-op and a two-drug (HR) regime post-op (Sandher et al., 2007; Lesić et al., 2010).

### 3.4 Surgical treatment

Surgery was performed on at least some of the patients in all of the included studies. For 81 patients, the only surgical procedure was a biopsy for diagnostic purposes (Qian et al., 2018; Sandher et al., 2007; Gursu et al., 2014; Kapukaya et al., 2006). Curative surgical interventions varied, ranging from debridement to total joint arthroplasty. The overall surgical rate was 82 % (367 of 477 patients) in those studies that reported this information explicitly. When arthroplasty and arthrodesis are excluded, 86 of 107 patients (80 %) received an open or arthroscopic debridement (Huang et al., 2007; Titov et al., 2004; Al-Sayyad and Abumunaser, 2011; Samuel et al., 2011; Duan and Yang, 2019; Kapukaya et al., 2006).

The studies involving TB of the hip joint had a total of 193 patients with a 100 % surgical intervention rate, 91 % being total hip arthroplasty (THA) and the remaining 9 % being hip arthrodesis (Kumar et al., 2015; Kim et al., 2001; Zeng et al., 2015; Liu et al., 2018). Uncemented implants were the most common prostheses used in 77 % (134 of 175) of cases. Kumar et al. (2015), Zeng et al. (2016) and Liu et al. (2018) collectively reported no osteolysis, stem subsidence nor cup loosening. Kumar et al. (2015) reported on 65 cases of TB hip. All patients completed 18 months of ATT and had proven resolution of active disease, both radiologically and biochemically, before they underwent uncemented total hip arthroplasty. No early or late surgical complications were recorded, but they did have two recurrences not requiring surgery. Zeng et al. (2015) also described no surgical complications in their cohort of 32 uncemented THA cases. Liu et al. (2018), who compared arthroplasty with arthrodesis in TB hip, echoed this finding in their arthroplasty group of 22 patients.

Similarly, all 58 patients with TB knee had surgery (Shen et al., 2010; Habaxi et al., 2014; Ozturkmen et al., 2014; Tang et al., 2015). Shen et al. (2010) reported 10 cases of early TB knee that all underwent arthroscopic debridement. No sinuses nor wound complications occurred, with all patients reported as having good outcomes and an increased range of knee motion. Habaxi et al. (2014) performed total knee arthroplasty (TKA) on 10 active cases of TB knee. Four patients had open sinuses at the time of recruitment, but all had closed sinuses at the time of surgery. No aseptic loosening, fractures nor dislocations occurred postoperatively. Ozturkmen et al. (2014) described 12 cases of TKAs in advanced TB knee; equivalently, they reported no pain, loosening nor any other surgical complications. The last study (Tang et al., 2015) looked at end-stage disease exclusively. All 26 patients underwent knee arthrodesis with cross-screws and a unilateral external fixator. Most (96 %) had fused successfully at 8 months post-op, and all had fused successfully at 14 months post-op. Complications included one patient who developed a sinus far from the site of surgery (greater trochanter area) that resolved with minor surgery and medical treatment alone.

In the studies looking at TB of the foot and/or ankle, the overall surgical intervention rate was 35 % (excluding biopsies) (Dhillon and Nagi, 2002; Tang et al., 2007; Samuel et al., 2011; Gursu et al., 2014; Duan and Yang, 2019). Surgical debridement was the most favoured procedure in 79 % of cases, and it was performed either open or arthroscopically. Dhillon and Nagi (2022) noted that only 5 out of 74 (7 %) of their patients required surgery: 3 had triple arthrodeses, 1 had a talonavicular fusion and 1 had two synovectomies. Two patients developed painful rocker bottom feet, and one patient had a painful spastic flat foot. Gursu et al. (2014) also performed a similar descriptive study. Here, 28 of 70 patients required additional procedures: 40 % underwent soft-tissue debridement, 34 % underwent bone debridement and 7 % underwent arthrodesis. At final follow-up, 56 % of patients had either severe bony destruction or end-stage arthrosis. In the cohort of Samuel et al. (2011), 7 out of the 16 (44 %) patients had surgery: 5 needed ankle fusion procedures and 2 required ankle synovectomies. Tang et al. (2007) looked at end-stage ankle TB cases that had arthroscopic joint preparation. All had fused at 14.5 weeks post-op using a ring fixator compression technique following joint preparation, and no complications were reported. Lastly, Duan et al. (2019) observed no adverse events in 15 cases of early-stage disease treated with arthroscopic debridement.

Kupukaya et al. (2006) analysed shoulder tuberculosis in 11 patients with a 27 % (3 of 11) surgical intervention rate. This comprised debridement in two cases and arthrodesis in the other. Pain-free shoulders were found at final follow-up.

A total of 6 of our 22 included studies comprised a mixture of involved joints (Qian et al., 2018; Titov et al., 2004; Huang et al., 2007; Al-Sayyad and Abumunaser, 2011; Sandher et al., 2007; Lesić et al., 2010). The mixed-cohort studies were largely comprised of spinal tuberculosis (39 %), followed by hip (14 %) and knee tuberculosis (11 %). These six studies had an overall surgical intervention rate of 44 % with an expected high heterogeneity due to the nature of this mixed cohort (Table 1).

**Table 4:** Consensus recommendations formulated by the EBJIS workgroup on tuberculous arthritis.

Research questions and recommendations		Grade of
		recommendations ${}^{*}$
When should the diagnosis of arthritis caused by *Mycobacterium tuberculosis* be suspected?		
	We recommend considering the diagnosis of tuberculosis (TB) arthritis in any patient with symptoms/signs of arthritis, such as joint pain, swelling, effusion, restricted movement and/or deformity involving one or more joints, with a subacute or chronic course (weeks to months or even years).	C1
	Additionally, we recommend increasing the suspicion of TB arthritis in the following cases:	
	– patients with a prior history of TB disease, simultaneous TB in other sites and/or chest radiography with signs of previous TB;	C1
	– patients presenting with monoarthritis with an indolent course or granulomatous inflammation, even if systemic symptoms such as low-grade fever, night sweats or weight loss are absent, and there are no evident risk factors for TB (e.g. migration from TB endemic area, comorbid illness and immunosuppression);	D1
	– any suspected infectious arthritis with conventional negative cultures and no response to empirical treatment of pyogenic arthritis;	D1
	– patients with arthritis and X-ray features of periarticular osteopenia, subchondral erosions, periarticular cysts or fractures, narrowing of the joint space, and/or bony destruction or sclerosis.	D2
What tests should be performed in patients with suspicion of native joint infection caused by *M. tuberculosis*?		
	We recommend sending for the following laboratory analyses:	
	– synovial biopsy specimen for acid-fast bacilli (AFB) stain, mycobacterial culture and nucleic acid amplification test (NAAT) as well as histopathological analysis;	C1
	– Sample of synovial fluid, with as much fluid as possible, in a sterile container for AFB stain, mycobacterial culture and NAAT.	C1
	We also suggest the following additional investigation:	
	– routine infection markers – erythrocyte sedimentation rate (ESR) > 40 mm h ${}^{-1}$ should raise suspicion;	C1
	– plain radiographs of the involved and contralateral joint are recommended in all patients at baseline (Although X-ray findings are often non-specific, they can serve as baseline image for evaluating differential diagnosis and any future joint impairment.);	C1
	– ultrasound can be useful for the detection and guided aspiration of joint fluid and periarticular abscess formation;	C1
	– magnetic resonance imaging is suggested if advanced imaging is required, such as in patients with suspicion of coexistent periarticular osteomyelitis or to better evaluate the extent of disease. It may also be useful when TB arthritis is suspected, and plain radiographs are normal (in the early stages of disease).	D2
What is the recommended antimicrobial therapy for arthritis due to *M. tuberculosis* and its optimal duration?		
	– Initial medical therapy for TB consists of a combination of four drugs including rifampin, isoniazid, ethambutol and pyrazinamide for 2 months; ethambutol may be discontinued if susceptibility to the other three drugs is demonstrated.	C1
	– After the 2-month induction period, patients with drug-susceptible TB should continue with isoniazid and rifampin.	C1
	– We recommend a minimum of a 6-month regimen for drug-susceptible TB.	D1
	– The treatment of patients with drug-resistant TB should be guided by an infectious diseases expert in the field.	D1
What are the special considerations related to the surgical management of native joint infection caused by *M. tuberculosis?*		
	– We recommend treating early cases of TB arthritis with medical therapy alone.	D2
	– Surgical intervention with debridement and synovectomy in the active phase of TB arthritis should be considered for patients with large abscesses, significantly devitalized bone or showing inadequate response to medical management.	C1
	– Patients with substantial joint destruction, ankylosis, deformity, significant loss of function or chronic pain after TB arthritis may benefit from operative management with excisional arthroplasty or arthrodesis.	D1
	– Total joint arthroplasty may be considered in patients with hip or knee involvement, but the optimal time to perform this type of surgery it is not clear.	D2

${}^{*}$ The GRADE (Grading of Recommendations, Assessment, Development and Evaluations) quality of evidence is presented as follows: A – high, B – moderate, C – low, and D – very low. The associated strength of recommendation is presented as follows: 1 – strong recommendation for or against an intervention and 2 – weak recommendation for or against an intervention.

### 3.5 Outcomes

The combined mean follow-up time of all studies was 26 months, with most demonstrating a minimum follow-up time of at least 6 months (range of 3–112 months). Recurrence rates were reported in most studies, with an overall average recurrence rate of 7.4 % (35 of 475). The average recurrence rate was 11.9 % in those studies that did observe cases of recurrence (35 of 252) (Kim et al., 2001; Kumar et al., 2015; Habaxi et al., 2014; Tang et al., 2015). A total of 10 studies reported no recurrences (0 of 223 patients) (Kapukaya et al., 2006; Zeng et al., 2015; Shen et al., 2010; Ozturkmen et al., 2014). It is not clear if there is any association between the duration of ATT and the rate of recurrence.

Only one study highlighted specific factors related to recurrence (Huang et al., 2007). The presence of multi-focal arthritis, drug-resistant *M. tuberculosis* strains and poor compliance with treatment were deemed to be the lead causative factors. The timeframe of disease recurrence was noted in two studies. The mean time to recurrence was 6 months, ranging from 3 to 9 months following completion of treatment (Huang et al., 2007; Habaxi et al., 2014). These recurrences were recognized by a combination of clinical features (presence of a new sinus or ongoing pain and swelling) and elevated inflammatory markers (CRP and ESR). The combined recurrence rate in the TB hip group was 4 %. Kim et al. (2001) stated that their 9 % recurrence rate was mostly due to improper chemotherapy administration due to interdepartmental communication errors or primary drug resistance. Kumar et al. (2015) encountered a 3 % recurrence with a 6-month treatment duration (2 months of HREZ followed by 4 months of HR), whereas Zeng et al. (2015) and Liu et al. (2018) observed no reactivation following 12 months of postoperative treatment consisting of four agents (HREZ/HRES). The recurrence rate in TB of the knee ranged from 0 to 4 % (Shen et al., 2010; Habaxi et al., 2014; Ozturkmen et al., 2014; Tang et al., 2015). The highest recurrence rate of 26 % was reported in the foot and ankle group (Gursu et al., 2014). Neither Tang et al. (2007) nor Duan et al. (2019) reported recurrences, while the other studies in the foot and ankle group did not specifically report on their recurrence rates. In terms of recurrence rates in the mixed-cohort group, Titov et al. (2004) and Sandher et al. (2007) found no recurrences. Qian et al. (2018) reported an overall cure rate of 82 %. Lešić et al. (2010) reported favourable outcomes in 94 % of cases. These two studies were on mixed cohorts, and the outcome measures were not explicitly defined. Dhillon and Nagi (2002) proposed the following criteria for a successful outcome and healing in foot and ankle TB: (1) resolution of local symptoms such as pain, swelling and healing of the sinuses; (2) decrease in the serial ESRs; and (3) radiologic evidence showing remineralization, decrease in osteoporosis and fusion in patients in whom arthrodesis was done.

Some studies also reported the functional outcomes or the outcomes of surgery. Titov et al. (2004), for example, reported significant functional improvement after arthroscopic debridement of the knee in terms of the range of motion (ROM) and Knee Scoring System. Similarly, Shen et al. (2010) used the ROM and the Japanese Orthopaedic Association knee rating system. Kapukaya et al. (2006) reported outcomes in terms of residual pain and ROM following treatment. Tang et al. (2007, 2015) and Samuel et al. (2011) reported a 100 % fusion rate of ankle and knee arthrodesis, while Liu et al. (2018) observed one non-union and one delayed union out of their 18 hip arthrodesis cases. Two series reported a 0 % implant loosening of hip and knee arthroplasty at a mean follow-up of 8 and 6 years, respectively (Kumar et al., 2015; Ozturkmen et al., 2014). We could not ascertain if surgical debridement plus ATT had favourable outcomes compared with ATT alone. The lack of data, variation with respect to methodology and lack of specific comparative studies focusing on this precluded it.

## 4 Discussion

The aim of this systematic review was to assess the available evidence on the diagnosis and management of tuberculous arthritis of native joints. The data emanating from this systematic review and the opinion of experts were then used to inform the international workgroup's recommendations. With regards to the specific research questions, the workgroup's consensus recommendations are provided in Table 4.

One of the main challenges in joint TB is the diagnosis, particularly because of its low incidence (especially in non-endemic areas), the usual absence of simultaneous lung disease and constitutional TB symptoms, and its indolent course with scarce inflammatory signs. TB arthritis should be considered in any patient presenting with a chronic granulomatous monoarthritis of insidious onset (Hogan et al., 2017; Berney et al., 1972). Previous history of tuberculosis (present in < 25 % of cases) and chest X-ray changes suggestive of active or old pulmonary tuberculosis (present in < 50 % of cases) should increase the suspicion of TB arthritis, even though these features are not ubiquitous (Mariconda et al., 2007; Huang et al., 2007). An ESR > 40 mm h ${}^{-1}$ should also raise suspicion (Mariconda et al., 2007; Huang et al., 2007; Dhillon and Nagi, 2002; Zeng et al., 2015). Imaging modalities, including routine radiography, ultrasound, CT and MRI, are useful adjuncts to the clinical work-up (Venkat et al., 2018). With these clinical findings being relatively non-specific, it is important to confirm the clinical suspicion of TB with an accurate and reliable diagnostic test. Our results suggest considerable variation in the way the diagnosis was made in the literature, and there is a need for standardization of the diagnostic criteria. AFB smears of synovial fluid have a low yield and are of little diagnostic value (Berney et al., 1972). Similarly, joint fluid cultures may only be positive in 25 % of cases (Allali et al., 2005). In most of the series in this systematic review, the diagnosis was made by histological analysis and/or microbiological culture from tissue obtained through biopsy. Histological evaluation and AFB smears are, however, currently not considered to be stand-alone definitive confirmatory tests, as they may be positive in non-tuberculous mycobacterial infections. A recent meta-analysis looking at the value of IGRAs performed on whole blood found a pooled sensitivity of 84 % and a specificity of 78 %. The authors concluded that IGRA on whole blood exhibits poor diagnostic accuracy in osteoarticular TB (Ren et al., 2022). The gold standard for diagnosis is currently considered to be the identification or isolation of *M. tuberculosis* by culture or NAAT, such as PCR (Hogan et al., 2017; Lewinsohn et al., 2017). Mycobacterial susceptibility study should always be performed on culture isolates. The diagnosis of TB arthritis often requires the microbiological study of synovial tissue, which is more sensitive (90 % of positive cultures) than synovial fluid analysis alone (Hogan et al., 2017; Bennet et al., 2020). Additional sampling of periarticular fluid collections may also be of value. Microbiological culture has traditionally been considered the most sensitive test, but its main drawback is the prolonged time (generally several weeks) it takes and low yield rates (Agashe et al., 2020). Alternative culture methods, like the BACTEC Myco/F lytic and MGIT960 systems, appear to be superior to Löwenstein–Jensen medium for the culture of body fluid and tissue samples (Wang et al., 2016; Zhao et al., 2023). However, limited data are available on the use of these media for articular TB specifically. NAATs can provide a rapid diagnosis; the Xpert MTB/RIF assay is an automated NAAT that can simultaneously detect *M. tuberculosis* and rifampin resistance, and it has also demonstrated good diagnostic accuracy for bone and joint TB (Shen et al., 2010; Li et al., 2018; Gu et al., 2015). This test, however, is not currently approved by the FDA (Food and Drug Administration) for samples other than sputum in the United States. Furthermore, the processing of osteoarticular specimens for PCR is important, and a standardized approach would be beneficial when comparing findings. Current data suggest that the more recently developed Xpert Ultra may increase the yield (Sun et al., 2019). Although a positive NAAT confirms the diagnosis, a negative result cannot rule out TB. Few data are available on the sensitivity and specificity of NAAT in the setting of culture-negative TB arthritis (Hogan et al., 2017).

Antimicrobial therapy remains the cornerstone of treatment. While some cases may be managed with medical therapy alone, surgical intervention may also be warranted (Hogan et al., 2017; Pigrau-Serrallach and Rodríguez-Pardo, 2013; Shen et al., 2010; Tuli, 2002). In early cases, ATT can result in complete resolution, without residual joint disease (Pigrau-Serrallach and Rodríguez-Pardo, 2013). This supports the importance of a high level of suspicion of joint TB and an early, aggressive approach to diagnosis. According to the Centers for Diseases Control and Prevention (CDC) guidelines, a 6- to 9-month regimen containing rifampin is recommended for the treatment of bone and joint TB (Nahid et al., 2016). Initial medical therapy for drug-susceptible TB consists of a combination of four drugs, including rifampin, isoniazid, ethambutol and pyrazinamide, for 2 months (ethambutol may be discontinued if susceptibility to the other three drugs is demonstrated) (Hogan et al., 2017; Nahid et al., 2016). After this induction period, patients with drug-susceptible TB should continue with isoniazid and rifampin for a minimum of 4 to 7 more months of therapy (Nahid et al., 2016). However, the optimal duration of ATT for bone and joint TB remains uncertain (Shen et al., 2010). Most of the information comes from older studies on spinal TB, with various limitations (Anonymous, 1986, 1993; Darbyshire, 1999). Our systematic review revealed considerable variation in the approach to medical management, with the duration of preoperative treatment varying from 2 to 12 weeks and postoperative treatment consisting of two to four drugs for anything from 4 to 18 months. Some experts tend to favour 9- to 12-month duration regimens, particularly in patients who present with a significant burden of disease or a high net state of immunosuppression (Shen et al., 2010). ATT should ideally be supervised by an expert in the field, particularly in the case of multidrug-resistant TB (MDR-TB). In the case of MDR-TB, the Infectious Diseases Society of North America (IDSA) recommends an intensive phase duration of 5–7 months (five drugs) and a total duration of 15–20 months following culture conversion (with four drugs for the continuation phase) (Nahid et al., 2019).

While the backbone of treatment of joint TB remains ATT, surgery is necessary in some cases, although its role in not always clear (Tuli, 2002). Surgical intervention in the active phase of TB arthritis is usually considered for patients with large abscesses, significant devitalized bone and/or those showing an inadequate response to medical management (Tuli, 2002; Lesić et al., 2010; Gursu et al., 2014). However, some authors have suggested that surgical debridement and synovectomy may expedite healing and limit joint damage (Vohra et al., 1997; Dhillon and Nagi, 2002; Samuel et al., 2011; Duan and Yang, 2019; Gardam and Lim, 2005). There is, however, little evidence in the literature to support this premise, as clinical trials or even sound observational studies with control groups are lacking. Further studies would be needed to determine if the addition of arthroscopic or open debridement is superior to medical management alone. Although total joint arthroplasty is typically not performed during the active phase of TB arthritis, it may be unavoidable in certain instances. In these cases, arthroplasty followed by long-term ATT may be used with some success (Hogan et al., 2017). While active infection was previously considered to be a contraindication for arthroplasty surgery, with long intervals of at least 10 years being advocated, there have been a number of reports of total hip arthroplasty (THA) in cases with active TB infection (Tuli, 2002; Duan and Yang, 2019). A previous systematic review on the outcome of single-stage THA in 65 patients with active hip TB found only one case of reactivation in a patient who was non-compliant with treatment after a mean follow-up of 53 months (2–9 years) (Kim et al., 2013). The evidence for TKA in patients with active knee TB, however, remains limited (Habaxi et al., 2014). Most authors emphasize the importance of a thorough debridement when doing arthroplasty in the setting of active infection. Zeng et al. (2016) performed a two-stage TKA following 3 months of preoperative ATT in four cases with active infection. At a mean follow-up of 4 years (range of 2–7 years), there was no reactivation of infections. Patients with quiescent infection and substantial joint destruction, fibrous ankylosis with significant loss of function and/or chronic pain after TB arthritis may also benefit from operative management (Hogan et al., 2017; Tuli, 2002). Excisional arthroplasty, joint replacement surgery or arthrodesis may be considered to treat these sequalae of TB arthritis (Hogan et al., 2017; Dhillon and Nagi, 2002; Tuli, 2002). For the hip and knee, joint replacement appears to be superior in terms of the functional outcome (Liu et al., 2018; Kumar et al., 2015; Zeng et al., 2015, 2016). Optimally, total joint arthroplasty after TB arthritis should be deferred until patients show no evidence of recurrent disease after completion of therapy, given the potential for reactivation disease after arthroplasty (Hogan et al., 2017). However, the optimal time interval between the treatment of joint TB and the arthroplasty surgery is not known. In most cases, patients undergoing joint replacement received prolonged pre- and postoperative ATT.

There are a number of limitations to this study. Firstly, the search terms were specifically chosen to yield as many data sources as possible. Despite this, articles focusing on certain specific aspects of joint TB may not have been included. For example, studies comparing the accuracy of the various diagnostic modalities of TB were not included. Secondly, with the focus being tuberculosis of the native joint, we may have missed data from studies looking at bone TB that included cases with joint involvement. We excluded case reports and small case series with less than 10 patients as well as papers from before 1970. In studies looking at a mixed cohort of cases, the diagnosis, management and outcomes were often not specifically reported per joint site, making any quantitative analysis problematic. There were few comparative studies; therefore, it was not possible to make definitive recommendations supported by high-level evidence. The lack of comparative studies focusing on the outcomes of surgical and non-surgical treatment hampered our ability to explore this subject any further. There is a dearth of data providing insight into the factors associated with an increased risk of the recurrence of infection. In the same vein, it remains unclear if the duration of ATT has a role to play in decreasing recurrence rates. If anything, this study has highlighted the great need for well-designed prospective cohort studies or randomized trials to shed more light on the many questions that remain.

## 5 Conclusions

The current literature on TB arthritis highlights the need for the establishment of standardized diagnostic criteria. Further research is needed to define the optimal approach to medical and surgical treatment and the timing and technique of reconstruction procedures. The role of early debridement in active TB arthritis needs to be explored further. Specifically, comparative studies are required to address the questions around the use of medical treatment alone vs. in combination with surgical intervention.

## Data Availability

The datasets generated during and/or analysed during the current study are available from the corresponding author upon reasonable request.
